# The Effectiveness and Complication Rate of Resorbable Biopolymers in Oral Surgery: A Systematic Review

**DOI:** 10.3390/dj13060264

**Published:** 2025-06-13

**Authors:** Riccardo Fabozzi, Francesco Bianchetti, Domenico Baldi, Catherine Yumang Sanchez, Francesco Bagnasco, Nicola De Angelis

**Affiliations:** 1 Department of Surgical Sciences and Integrated Diagnostics, University of Genoa, L. go R. Benzi 10, 16121 Genoa, Italy; 5138519@studenti.unige.it (R.F.); 5410011@studenti.unige.it (F.B.); domenico.baldi@unige.it (D.B.); francesco.bagnasco@edu.unige.it (F.B.); 2 Private Practice, Corso Bagni 54, 15011 Acqui Terme, Italy; drcatyumang06@gmail.com

**Keywords:** biocompatible materials, bone regeneration, biopolymers, oral surgical procedures, polyesters, tissue engineering

## Abstract

**Background**: Resorbable biopolymers are increasingly explored for use in regenerative procedures within dental surgery. Their ability to degrade naturally, minimize surgical reinterventions, and potentially reduce immunogenicity makes them appealing in guided bone and tissue regeneration applications. However, despite these advantages, uncertainties persist regarding their comparative effectiveness and associated risks. For example, polyethylene glycol (PEG)-based membranes have shown comparable outcomes to porcine-derived collagen membranes in bone regeneration procedures, yet studies have reported a higher incidence of soft tissue healing complications associated with PEG-based materials. Similarly, while polycaprolactone (PCL) and dextrin-based hydrogels have demonstrated promising clinical handling and bone fill capabilities, their long-term performance and consistency across different anatomical sites remain under investigation. These findings highlight the need for further well-powered clinical trials to establish standardized guidelines for their safe and effective use. **Methods**: A systematic review protocol was registered with the PROSPERO database and developed in alignment with PRISMA guidelines. Database searches were conducted in PubMed, Medline, Scopus, and Cochrane from June to December 2024. Only randomized controlled trials (RCTs) focusing on synthetic resorbable biopolymers in bone augmentation procedures were considered. Bias was evaluated using the Cochrane Risk of Bias tool. **Results**: Eleven RCTs were included, totaling 188 patients. The findings suggest that materials such as polylactic acid (PLA), polycaprolactone (PCL), and polyethylene glycol (PEG) contributed effectively to new bone formation. PEG-based membranes were found to perform on par with or occasionally better than traditional collagen membranes derived from porcine sources. Additionally, the application of 3D-printable polymers demonstrated promise in site-specific healing. **Conclusions**: Resorbable biopolymers are effective and safe for GBR procedures, with clinical outcomes comparable to traditional materials. Advances in 3D-printing technology and bioactive coatings may further enhance their regenerative potential. However, the incidence of soft tissue healing complications suggests the need for further long-term studies to optimize material properties and clinical application.

## 1. Introduction

In modern dentistry, the increasing prevalence of conditions affecting the oral cavity necessitates the use of regenerative medicine. This approach focuses on restoring lost tissues to their original anatomy, functionality, and aesthetics.

Various factors can contribute to bone defects in the maxillae, including periodontal disease, trauma, surgical resection due to neoplasms, and tooth extractions [1,2]. If left untreated, these conditions may lead to atrophy of the maxillary bones. Tooth extraction, in particular, can result in significant bone resorption within the first six months, reducing bone availability for subsequent rehabilitation with conventional prostheses or dental implants [3,4,5].

To address bone defects in the maxillae, guided bone regeneration (GBR) or guided tissue regeneration (GTR) techniques are often employed [6,7,8].

These approaches involve using resorbable or non-resorbable membranes positioned coronally to the defect. The membranes isolate the defect, prevent invasion by surrounding connective tissue, and create a space conducive to new bone growth [6,7,8,9,10,11].

Regenerative materials include not only membranes but also bone grafts, which may be autogenous (harvested from the patient), allogeneic (harvested from a donor, typically cadaveric), or xenogeneic (harvested from a different species, such as bovine) [12,13,14].

These materials can be classified as follows:

- Osteoconductive: Serving as a scaffold for new bone growth, guiding the regenerative processes of native bone.
- Osteoinductive: Stimulating the formation of new osteoblasts to accelerate regeneration.
- Osteopromotive: Enhancing the osteoinductive effect of other grafts or membranes [15].

Regenerative branches of dentistry have led to the development and clinical application of conductive and inductive biomaterials used in sinus augmentation, ridge preservation, guided bone regeneration (GBR), and periodontal regenerative procedures. A notable example includes the use of autologous platelet-rich fibrin (PRF) and collagen membranes in combination with bone grafts for the treatment of Grade II furcation defects, as demonstrated in a randomized controlled study comparing their clinical efficacy [16].

Among osteoconductive materials, hydroxyapatite, tricalcium phosphate (TCP), and various polymers—such as polylactic acid, polyglycolic acid, poly-ε-caprolactone, and their copolymers (collectively known as aliphatic polyesters)—are widely used for bone regeneration [1,17].

Osteoinductive materials include platelet-rich growth factors (PRGF), platelet-rich plasma (PRP), and platelet-rich fibrin (PRF) [18].

Cellulose-based materials have also demonstrated osteoconductive properties, but to fully express their osteoinductive potential, they must often be modified or combined with other bioactive materials. This represents a key area of current research in bone tissue engineering [19].

Other materials, such as ABM/P-15, have shown the ability to enhance periodontal ligament (PDL) regeneration by modulating the expression of specific cellular genes during early wound healing [20].

The combination of these materials, chosen according to the nature of the defect and patient-specific needs, represents a major innovation in regenerative dental surgery, contributing to increasingly predictable and satisfactory clinical outcomes.

This review focuses on resorbable membranes, an evolution of first-generation non-resorbable membranes. Resorbable membranes offer significant advantages, including the elimination of a second surgery for removal [21], reduced patient morbidity, simplified surgical procedures, and a lower risk of membrane exposure [22].

Resorbable membranes, or second-generation membranes, were developed to avoid the need for a second surgical intervention while offering lower costs and a reduced risk of complications. Initially, these membranes were made from natural polymers like type I and III collagen. Advances in technology have led to the development of synthetic membranes, such as biodegradable aliphatic polyesters. For synthetic membranes, combining them with bone substitute materials is crucial to fill bone defects, thereby creating and maintaining the necessary space for new bone formation [23].

Bioresorbable membranes, particularly those composed of type I and III collagen, have been widely adopted in regenerative dental procedures due to their biocompatibility and ability to integrate with host tissues. These membranes are typically derived from natural sources such as porcine, bovine, or human pericardium, dermis, or tendon, and undergo decellularization processes that preserve their three-dimensional collagen network. Studies have shown that these membranes not only act as passive barriers but also actively modulate cell behavior. In vitro experiments with bovine pericardium membranes have demonstrated that human periodontal ligament fibroblasts (HPLFs) exhibit enhanced adhesion, spreading, and proliferation on their surfaces, forming cellular extensions like lamellipodia and filopodia to bind with the membrane fibers [24]. In vivo, collagen membranes are enzymatically resorbed by matrix metalloproteinases (MMPs) released by neutrophils, monocytes, and fibroblasts, while simultaneously supporting new connective tissue attachment and preventing epithelial migration, as initially observed in canine models. These properties highlight the dual mechanical and biofunctional role of collagen-based membranes in guided bone and tissue regeneration.

Non-resorbable membranes, such as those made of titanium, remain widely used but are associated with higher costs compared to more commonly used materials. Non-resorbable synthetic polymer membranes, like expanded polytetrafluoroethylene (e-PTFE), are also prevalent but exhibit a high rate of exposure. Additionally, non-resorbable silk membranes are challenging to handle and have poor mechanical properties [25,26].

The latest innovation involves 3D-printed membranes, leveraging technology widely applied in fields such as anatomical modeling, surgical guide production, and regenerative medicine. These membranes incorporate biomaterials, including hydrogels combined with live cells and/or growth factors, natural and synthetic bioplastics, proteins, polymeric biomolecules, and ceramics [27].

The aim of the present systematic review is to assess whether resorbable synthetic biopolymers are safely employed in oral regenerative surgery and if there are evidences reporting the complication rate with this purpose.

## 2. Materials and Methods

### 2.1. Search Strategy

This systematic review was registered on the PROSPERO database with the ID 1043600 and is available at the following link: https://www.crd.york.ac.uk/PROSPERO/view/CRD420251043636 (accessed on 30 April 2025).

The study was conducted using PubMed, Medline, Scopus, and Cochrane databases from June to December 2024; however, no articles were retrieved from the latter. This outcome was not due to limitations in the search strategy, which was applied consistently across all databases, but rather to the absence of randomized controlled trials meeting the inclusion criteria within the Cochrane Library at the time of the search.

The initial keywords used were “Biopolymers and Regenerative Dental Surgery” and “Biopolymers and Guided Bone Augmentation”. These were subsequently rearranged to perform a Boolean search: “Regenerative Dental Surgery and Biopolymers” and “Guided Bone Augmentation and Biopolymers”. No time or language restrictions were applied during the search.

In April 2025, the search strategy was expanded by incorporating two new keyword combinations: “Biopolymer Synthetic Dentistry” and “Synthetic Guide Bone Augmentation”, which were similarly rearranged for Boolean search as “Synthetic Biopolymer Dentistry” and “Guide Bone Augmentation Synthetic”. For these searches, a filter was applied to include only randomized controlled trials (RCTs) published within the past 10 years.

This review aimed to address the following focused questions: *Are resorbable biopolymers used for regenerative surgery? Are resorbable biopolymers performing better than conventional materials? Are there any complications reported?* Following the PICO structure:

- **POPULATION:** Patients requiring a bone augmentation procedure for implant dentistry.
- **INTERVENTION:** Bone augmentation using synthetic resorbable polymers.
- **COMPARISON:** Comparison among different types of resorbable polymers.
- **OUTCOME:** Effective bone augmentation (with evidence of effective volume measures in the interval time included) and reported complications related to the procedure.

The studies to be included in this systematic review had to meet the following criteria:

- Human RCT studies focusing on regenerative surgery with at least a minimum number of 10 subjects included.
- No restriction for the anatomical site.
- Use of synthetic biopolymers.

Exclusion criteria were referred to all the other conditions not mentioned above.

The selection of studies was conducted following the PRISMA (Preferred Reporting Items for Systematic Reviews and Meta-Analyses) guidelines. The process involved the removal of duplicates, screening titles and abstracts, and a full-text assessment of potentially eligible studies. Reasons for exclusion at each stage were documented.

All outcomes for which data were sought were clearly defined a priori, and for each outcome domain, all compatible results reported within each study—including different measures, time points, and analyzes—were considered; in instances where multiple results were available, predefined criteria such as clinical relevance, consistency across studies, and longest follow-up were used to determine which data to extract.

### 2.2. Risk of Bias Assessment

The risk of bias for each included study was assessed independently for each domain, following the Cochrane Risk of Bias Tool. For every domain (e.g., random sequence generation, allocation concealment, blinding, incomplete outcome data, and selective reporting), specific criteria were applied to classify the risk as low, unclear, or high. A domain was rated as low risk if adequate methodological details were clearly reported and appropriate techniques were used (e.g., computer-generated randomization and allocation concealment through opaque envelopes). It was rated as unclear risk if reporting was insufficient or lacked detail to allow judgment, and as high risk when the methods used were inadequate or clearly introduced bias (e.g., lack of blinding in outcomes likely to be influenced by knowledge of intervention).

Two independent reviewers (CSY and DB) conducted the risk of bias assessment. Any discrepancies were resolved through discussion or by consulting a third reviewer (MM).

## 3. Results

A total of 648 studies were initially identified through the literature search. After the removal of duplicates and exclusion of articles that did not meet the predefined inclusion criteria, 11 studies were considered eligible for inclusion in this review.

The selected studies include the randomized clinical trial by Shahdad et al. [28], which compared a PEG-based synthetic membrane to a porcine-derived collagen membrane in the preservation of alveolar bone following tooth extraction in the anterior maxilla (published in 2020 in Clinical Oral Implants Research). Jung et al. [29] conducted a randomized, controlled clinical trial evaluating a novel membrane for guided bone regeneration around dental implants (published in Clinical Oral Implants Research in 2009). Similarly, Zwahlen et al. [4] compared two resorbable membrane systems for bone regeneration following the extraction of wisdom teeth in a randomized controlled clinical pilot study, reported in the same journal in 2009.

De Angelis et al. [30] investigated the soft tissue response to 3D-printable biopolymers used in socket preservation in a pilot randomized clinical study, published in 2024 in Dent J (Basel). Saini et al. [31] assessed the efficacy of combination techniques in enhancing the regenerative potential of tricalcium phosphate grafts in the treatment of infrabony periodontal defects, in a 2011 study published in the Indian Journal of Dental Research. Pradeep et al. [32], in a 2009 article from the Journal of Periodontology, evaluated the clinical effectiveness of autologous platelet-rich plasma combined with a Peptide-enhanced bone graft in the management of intrabony defects.

Machado et al. [33] conducted a randomized clinical trial on the use of an injectable dextrin-based hydrogel as a carrier for a synthetic bone substitute, with results published in 2023 in Clinical Oral Investigations. Kusirisin et al. [34], in a 2023 study in Clinical Implant Dentistry and Related Research, compared polycaprolactone and collagen membranes in terms of 1-year clinical outcomes. In a multicenter clinical trial, Jung et al. [35] compared a polyethylene glycol membrane to a collagen membrane for the treatment of bone dehiscence defects at bone-level implants, published in Clinical Oral Implants Research in 2020.

Additionally, Santana et al. [36] reported the outcomes of a randomized, controlled clinical and histological trial evaluating a synthetic polymeric barrier membrane in combination with blood coagulum, human allograft, or bovine bone substitute for ridge preservation, published in 2019 in the International Journal of Oral and Maxillofacial Surgery. Finally, Arunjaroensuk et al. [37] investigated the stability of augmented bone when using two different membranes for guided bone regeneration performed simultaneously with dental implant placement in the esthetic zone, as published in the International Journal of Oral & Maxillofacial Implants in 2018.

**Figure 1:**
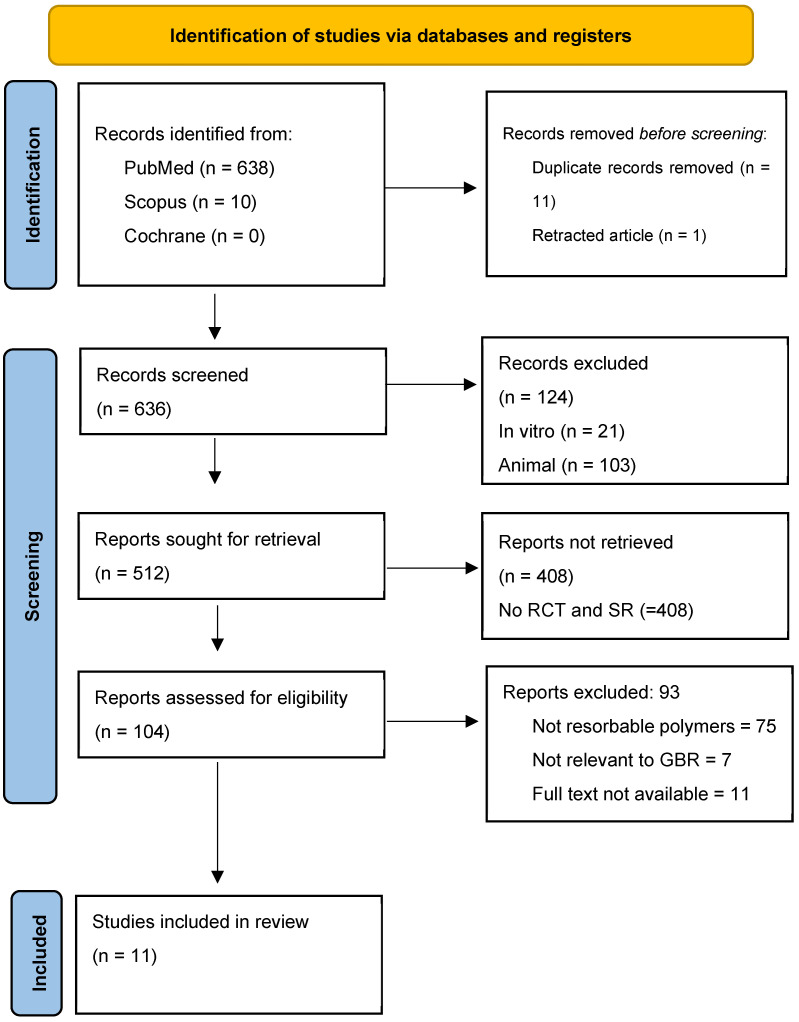
PRISMA 2020 flow diagram for new systematic reviews which included searches of databases and registers only.

A PRISMA flow diagram (Figure 1) is provided to illustrate the study selection process, including the number of records identified, screened, excluded, and included in the final review.

A summary of the included studies, including their main objectives, methods, and findings, is provided in Table 1. These studies were selected based on the inclusion criteria described above.

This systematic review was conducted without a meta-analysis, primarily due to the high methodological heterogeneity observed among the included studies. The differences mainly concerned the experimental designs, which ranged from single-center pilot studies to randomized controlled multicenter trials, with varying levels of randomization, blinding, and allocation methods. Additionally, the characteristics of the studied populations differed in terms of patient numbers, age, treated anatomical sites (mandible vs. maxilla; anterior vs. posterior regions), and specific clinical indications (e.g., site preservation, horizontal or vertical regeneration, and peri-implant defects). Outcome measures were also highly heterogeneous: some studies evaluated clinical parameters (such as probing depth or primary implant stability), while others relied on radiographic or histological data (such as the percentage of new bone formation or material resorption), with follow-up periods ranging from 6 weeks to 18 months. These differences in assessment criteria, observation times, and data collection methods would have compromised the reliability of a quantitative synthesis. Therefore, a qualitative synthesis of the results was chosen, considered more appropriate to coherently and clinically interpret the available data.

**Figure 2:**
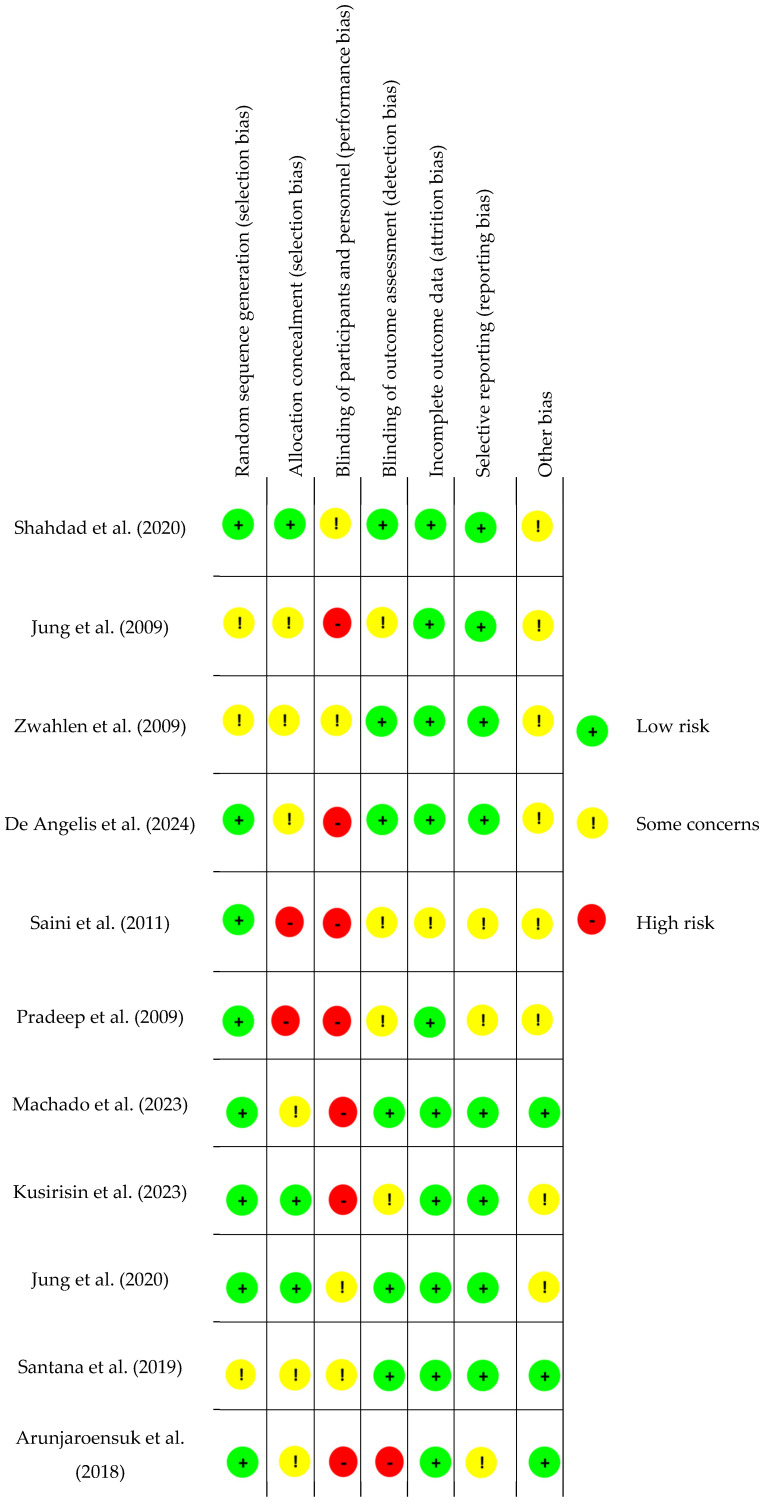
The risk of bias assessment of each included study [4,28,29,30,31,32,33,34,35,36,37].

All the studies were screened for bias assessment and in Figure 2 all the data are reported.

### 3.1. Are Resorbable Biopolymers Used for Regenerative Surgery?

Resorbable biopolymers have emerged as a valuable tool in regenerative surgery, particularly in bone regeneration and post-extraction socket preservation. Various studies have explored different materials, analyzing their efficacy in maintaining alveolar ridge dimensions and supporting new bone formation. De Angelis et al. [30] evaluated the effectiveness of 3D-printable biopolymers, specifically Poly-D-lactic acid (PDLA) with hydroxyapatite and Poly-ε-caprolactone (PCL) with β-tricalcium phosphate (β-TCP). These materials function as protective barriers that gradually degrade while promoting soft tissue healing and maintaining bone volume. Their study found that these biopolymers significantly enhanced healing in posterior extraction sites compared to spontaneous healing. Similarly, Zwahlen, Cheung, and Zheng et al. [4] compared the performance of a synthetic biodegradable membrane, the Inion GTR Biodegradable Membrane, composed of polylactides, polyglycolides, and trimethylene carbonate, with Geistlich Bio-Gide^®^, a porcine-derived collagen membrane. Their study found that both materials effectively facilitated bone regeneration, with no statistically significant differences in bone density between the two. This suggests that synthetic biodegradable membranes may offer a viable alternative to conventional animal-derived materials in guided bone regeneration. Another investigation conducted by Saini, Singh, Lal et al. [31] examined the role of tricalcium phosphate (TCP), a synthetic bone substitute, in combination with citric acid (CA) and oxidized regenerated cellulose (ORC), both of which serve as regenerative enhancers. Their findings highlighted the role of resorbable materials in improving bone healing, further demonstrating the potential of biopolymers in periodontal and bone defect treatments.

Machado A. et al. [33] carried out a clinical investigation involving 12 patients who required maxillary premolar extractions followed by ridge augmentation. The study compared two approaches: one group received only a synthetic granular bone graft (Bonelike^®^), while the other group received the same graft material combined with a dextrin-based hydrogel (DEXGEL Bone), forming an injectable, moldable composite. Overall, DEXGEL Bone demonstrated comparable or better outcomes than Bonelike^®^ alone, offering practical advantages such as improved surgical handling, enhanced implant stability, and quicker material resorption—without compromising safety or efficacy.

Kusirisin et al. [34] evaluated a novel bilayered polycaprolactone (PCL) membrane against a standard collagen membrane (Cytoplast™ RTM) in a randomized study with 24 patients undergoing guided bone regeneration (GBR) with simultaneous implant placement. The findings suggest that the bilayered PCL membrane performs on par with traditional collagen membranes in GBR, with similar esthetic and clinical outcomes, while offering the potential benefits of synthetic biomaterials.

Jung RE et al. [35] conducted a multicenter randomized trial with 117 participants to assess bone regeneration in dehiscence-type defects around implants. Subjects were divided into two cohorts: one treated with a polyethylene glycol (PEG) membrane and the other with a collagen-based (BG) membrane, both combined with biphasic calcium phosphate grafts. Findings suggested that PEG membranes support bone growth effectively, but their higher complication rate suggests a need for further research before broader clinical adoption.

Santana R. [36] evaluated the use of a PEG membrane paired with different grafting materials—blood clot (BC), human allograft (AL), and deproteinized bovine bone (BB)—in preserving the alveolar ridge post-extraction. Results showed that PEG membrane is effective as a barrier material.

Arunjaroensuk et al. [37] conducted a randomized clinical trial involving 60 implants placed with simultaneous GBR, comparing synthetic polylactic acid (PLA) membranes to traditional collagen membranes. Both groups used biphasic calcium phosphate as the graft material. The study concluded that PLA membranes are a viable alternative to collagen in GBR procedures, offering equivalent bone support, albeit with less intraoperative flexibility.

### 3.2. Are Resorbable Biopolymers Performing Better than Conventional Materials?

The performance of resorbable biopolymers compared to conventional materials varies depending on the clinical context and specific application. While some studies have reported advantages associated with biopolymer-based materials, others have found little to no significant difference in clinical outcomes. De Angelis et al. [30] observed that their 3D-printable biopolymers significantly improved healing in posterior teeth but did not offer substantial benefits in anterior sites, suggesting that their effectiveness may be site-specific. Their findings imply that while these materials may be beneficial for posterior alveolar ridge preservation, they might not be essential in areas with a less resorptive tendency. Similarly, Jung, Hälg, and Thoma et al. [29] investigated the use of polyethylene glycol (PEG)-based hydrogel membranes in bone defect treatments. Their study found that vertical defect fill was greater in sites treated with PEG-based membranes (5.63 mm) compared to sites treated with conventional collagen membranes (4.25 mm). However, the overall percentage of defect fill was nearly identical between the two groups, with the PEG group achieving a fill rate of 94.9% and the collagen membrane group achieving 96.4%. This suggests that while PEG membranes may provide a greater absolute defect fill, the overall regenerative capacity of both materials is comparable. Similarly, Zwahlen, Cheung, and Zheng et al. [4] found that synthetic membranes such as the Inion GTR system achieved bone density levels similar to those of the animal-derived Bio-Gide^®^ membrane. Their results indicate that synthetic biodegradable membranes perform equally well as traditional collagen membranes in terms of their barrier function and ability to support bone formation. Meanwhile, Saini, Singh, and Lal et al. [31] examined the effects of TCP combined with CA and ORC on bone defect healing. Their study revealed that the combination of TCP, CA, and ORC produced the highest mean values for defect fill and clinical attachment level gains when compared to TCP alone. However, the differences between the groups were not statistically significant, indicating that while the addition of CA and ORC showed some improvements, the impact was not substantial enough to be considered clinically superior. These findings collectively suggest that while resorbable biopolymers may offer advantages in specific scenarios—such as increased ease of handling, controlled degradation, and reduced immunological risks associated with animal-derived materials—their overall performance in terms of bone regeneration remains largely comparable to that of conventional materials.

Machado A. et al. [33] compared two approaches: one group received only a synthetic granular bone graft (Bonelike^®^), while the other group received the same graft material combined with a dextrin-based hydrogel (DEXGEL Bone), forming an injectable, moldable composite. Overall, DEXGEL Bone demonstrated comparable or better outcomes than Bonelike^®^ alone, offering practical advantages such as improved surgical handling, enhanced implant stability, and quicker material resorption—without compromising safety or efficacy.

### 3.3. Are There Any Complications Reported?

Although resorbable biopolymers have demonstrated promising clinical outcomes, some studies have reported complications associated with their use. Shahdad, Gamble, and Matani et al. [28] noted that while their study did not observe major complications, previous research had suggested that PEG-based membranes may be associated with delayed soft tissue healing. In their study, however, these complications did not negatively impact the overall regenerative outcome. Similarly, Jung, Hälg, and Thoma et al. [29] reported that PEG-based membranes had a higher incidence of mild to moderate complications compared to collagen membranes. Among these complications, delayed healing and dehiscence over the cover screw were the most commonly reported. Despite these occurrences, all sites eventually healed without long-term consequences. Zwahlen, Cheung, and Zheng et al. [4] documented several minor complications, including wound infections, hematomas, and delayed gingival swelling. Some cases of membrane exposure were observed, although these incidents did not interfere with the final bone regeneration outcome. Pradeep, Shetty, and Garg et al. [32] found no major complications in their study, although they acknowledged that platelet-rich plasma (PRP)-based therapies carry theoretical risks, including potential viral contamination (if allogeneic PRP is used), immune reactions to bovine thrombin, and a possible risk of exposure to variant Creutzfeldt–Jakob disease due to the use of bovine-derived products. De Angelis et al. [30] reported a complete absence of complications in their study, with no cases of infection, prolonged biomaterial exposure, or abnormal inflammatory responses. Their findings suggest that 3D-printable biopolymers are safe and well tolerated in clinical applications. Similarly, Saini, Singh, and Lal et al. [31] observed no significant adverse effects in their study, aside from mild inflammatory responses in some cases, which resolved without intervention. These reports indicate that while most resorbable biopolymers are well tolerated, certain materials—such as PEG-based membranes—may be associated with a slightly higher incidence of minor complications compared to conventional materials like collagen membranes.

Santana RG et al. [36] evaluated the use of a PEG membrane paired with different grafting materials and only minimal (not described) complications were reported.

Similarly, Arunjaroensuk et al. [37] compared synthetic polylactic acid (PLA) membranes to traditional collagen membranes. Minor complications were few and resolved spontaneously. While collagen membranes were easier to handle intraoperatively, PLA required more adaptation.

## 4. Discussion

The reviewed literature demonstrates that resorbable biopolymers represent a viable and increasingly studied class of materials in the context of regenerative surgery, particularly within the domain of guided bone regeneration (GBR) and socket preservation. Their biodegradability, customizable properties, and potential for avoiding the limitations associated with animal-derived products position them as attractive alternatives or adjuncts to conventional materials.

Several studies reported a comparable clinical performance between synthetic biopolymer-based membranes and widely used collagen-based counterparts. For instance, Jung et al. [29] and Zwahlen et al. [4] found that polyethylene glycol (PEG) and other synthetic membranes yielded similar regenerative outcomes to collagen membranes in terms of bone fill and density. However, in many of these studies, the differences between groups were not statistically significant, suggesting functional equivalence rather than a superiority of biopolymers. These results underscore that while synthetic materials can match conventional options in performance, they are not yet demonstrably superior across all contexts.

Site-specific efficacy appears to be a relevant consideration. De Angelis et al. [30] observed enhanced healing in posterior extraction sockets with 3D-printable biopolymer scaffolds, but similar benefits were not observed in anterior regions. This highlights the need to account for anatomical and biomechanical differences when selecting regenerative materials. Factors such as site resorption tendencies, surgical accessibility, and esthetic requirements should guide material choice; moreover, the integration of 3D-printed materials opens the door to fully personalized treatments, minimizing surgical complexity and potentially improving healing outcomes.

Material handling characteristics and surgical practicality also emerge as important differentiators. Dextrin-based hydrogels, such as DEXGEL Bone, demonstrated improved moldability and clinical handling compared to particulate bone grafts alone, without compromising efficacy (Machado et al. [33]). Such features may enhance clinician preference and patient outcomes, particularly in complex or confined surgical sites. Similarly, the bilayered PCL membrane evaluated by Kusirisin et al. [34] offers practical handling advantages over traditional membranes while achieving similar clinical results.

### Complications Associated with Resorbable Biopolymers

In terms of safety, the majority of studies reported minimal or no complications. PEG-based membranes, while effective, were occasionally associated with delayed healing or minor soft tissue complications (Jung et al. [29]; Shahdad et al. [28]). These events were generally self-limiting and did not compromise the overall regenerative success. Importantly, the absence of immunogenic reactions or adverse outcomes in most trials supports the biocompatibility of resorbable polymers when properly applied. Moreover, reports of successful application without inflammation or infection (e.g., De Angelis et al. [30]) lend confidence to their continued clinical use.

The combination of biopolymers with bioactive agents (e.g., tricalcium phosphate, citric acid, or oxidized regenerated cellulose) may offer additive benefits. Saini et al. [31] demonstrated slightly improved clinical attachment levels and defect fill with such combinations, although differences were not statistically significant. These data suggest a potential for optimization, albeit without definitive evidence of superiority.

Collectively, the findings reviewed support the use of resorbable biopolymers as effective and safe materials for GBR and related procedures. While they do not universally outperform conventional materials, they offer specific advantages—such as tunable degradation, reduced immunogenicity, and improved handling—that can be leveraged in clinical decision-making [23]. Further high-powered, long-term clinical trials are warranted to define optimal indications, cost-effectiveness, and comparative outcomes across diverse clinical scenarios. The evolution of biopolymer technologies may allow for increasingly tailored applications in regenerative therapy, aligning with precision medicine and biomaterial innovation [38].

From a translational perspective, the review points toward a future in which dental regenerative materials are selected not only for their mechanical and degradation properties but also for their compatibility with personalized regenerative strategies [38]. Advances in biomaterial research, including nanotechnology, surface modification, and smart material development [39], will likely converge with additive manufacturing and patient-specific planning to create an entirely new class of dynamic regenerative solutions. These innovations may soon permit clinicians to select or even fabricate scaffolds based on a patient’s age, metabolic rate, and tissue healing characteristics, enabling precision-guided tissue engineering [40,41,42,43].

The small number of studies included in this review limits the ability to assess the long-term outcomes of these materials, particularly beyond 6 to 12 months of follow-up. Longer-term evaluations are needed to confirm the stability of regenerated bone, the success rate of subsequent implant placements, and the potential for late-onset complications. Additionally, standardization in outcome reporting, membrane characterization, and surgical protocols would significantly enhance the comparability of future studies and facilitate meta-analytic evaluations.

A more critical look at the methodological limitations of the included studies reveals several recurring issues. Many trials enrolled small patient cohorts—often fewer than 30 per group—frequently without formal sample size calculations, thereby limiting statistical power and external validity. Follow-up durations were often short, ranging from just a few weeks to six months, making it difficult to evaluate long-term bone maturation and implant success. Moreover, there was a marked variability in outcome measures: while some studies used clinical indicators (e.g., probing depth and soft tissue healing), others relied on radiographic or histologic endpoints, which were not always comparable. This heterogeneity in study design and outcome assessment was a major reason for refraining from meta-analysis and continues to be a barrier to drawing robust conclusions. Future investigations should aim for larger sample sizes, longer observation periods, and more consistent reporting standards to improve the quality and translatability of the evidence.

Another important consideration is the potential impact of publication bias introduced by restricting inclusion to randomized controlled trials (RCTs). While RCTs are considered the gold standard for evaluating clinical efficacy, they often have strict inclusion criteria, relatively small sample sizes, and may not fully capture the variability of real-world clinical scenarios. As a result, excluding high-quality observational studies—particularly prospective cohort studies—might have led to the omission of relevant clinical data. During the screening process, several observational studies with robust methodologies were identified; however, they were excluded to preserve methodological consistency and internal validity. Future systematic reviews or meta-analyzes could consider including both RCTs and well-designed non-randomized studies to offer a more comprehensive overview of the clinical performance of resorbable biopolymers across diverse patient populations and treatment settings.

It is also essential to consider the economic and environmental implications associated with the adoption of synthetic resorbable biopolymers. Although these materials may currently be more expensive on a per-unit basis compared to traditional collagen membranes, their potential for in-house or chairside fabrication using 3D-printing technologies could significantly reduce overall procedural costs. This is particularly relevant in complex cases that would otherwise require multiple surgeries, custom grafts, or patient-specific devices. Additionally, the extended shelf life and reduced need for cold-chain storage of many synthetic polymers can lower logistical expenses and waste.

From an environmental standpoint, many biopolymers—including PCL and PLA—are derived from renewable resources such as corn starch or sugarcane, and are biodegradable, which contrasts with non-degradable synthetic materials or animal-derived products requiring more intensive processing. Their degradation byproducts are generally non-toxic and do not accumulate in tissues, which may also minimize the environmental burden associated with disposal. Furthermore, reducing the reliance on animal-derived materials may contribute to more ethical and sustainable clinical practice, aligning with global trends in green healthcare. However, detailed life-cycle assessments (LCAs) are still lacking in dental applications, and future studies should aim to quantify the full environmental impact of these materials from production to degradation.

Environmental sustainability is another factor gaining relevance in material science and clinical decision-making. Many of the polymers discussed—particularly PCL and PLA—are derived from renewable sources and are biodegradable, contributing positively to environmental objectives [44]. Future research may further explore the life-cycle analysis of these materials, considering not only clinical efficacy but also their ecological footprint.

In summary, although the materials explored in this review are not entirely new, their application in the context of modern surgical workflows and digital planning represents a pivotal advancement. The ability to design and fabricate custom, bioresorbable scaffolds marks a step forward in bridging conventional regenerative techniques with the broader movement toward personalized medicine. As the field continues to evolve, the convergence of digital technologies, advanced biomaterials, and molecular enhancements will likely define the next era of regenerative dentistry.

Therefore, while acknowledging the limitation posed by the inclusion of only a few studies, this review underscores the clinical relevance of resorbable biopolymers and introduces emerging technologies that may soon transform the landscape of dental regenerative procedures. These findings provide a valuable foundation for future investigations, particularly those focused on patient-centered approaches that integrate precision, efficiency, and biological synergy in tissue regeneration.

## 5. Conclusions

Overall, the data confirm the effectiveness of resorbable membranes and scaffolds in bone regeneration, with minimal differences between synthetic and natural materials. The use of emerging technologies, such as 3D printing and the combination of biomaterials with growth factors, represents a promising research field. However, the incidence of complications related to soft tissue healing and the variability of results depending on defect location suggest the need for further long-term clinical studies to optimize bone regeneration strategies in dentistry. Future research should also focus on enhancing the mechanical properties and degradation profiles of resorbable biopolymers to maximize their clinical effectiveness while minimizing complications. Additionally, the integration of nanotechnology and bioactive coatings in resorbable membranes may further enhance their regenerative potential, paving the way for more advanced and efficient GBR solutions in the future.

## Figures and Tables

**Table 1:** Main characteristics of the included studies.

Author	Article Title	Description	Number of Patients	Time of Follow Up	Key Findings	Complications Reported	Conclusions
Shahdad et al. [28]	Randomized clinical trial comparing PEG-based synthetic to porcine-derived collagen membrane in the preservation of alveolar bone following tooth extraction in anterior maxilla	The aim of this randomized controlled clinical trial was to compare alveolar bone preservation covered by either a synthetic membrane or a porcine collagen membrane.	30	6 months	PEG membrane resulted in significantly lower percentage loss in both labial and coronal dimensions compared to porcine collagen. Implant placement was comparable in both groups.	No major complications reported.	Use of PEG membrane led to a better preservation of ridge dimensions following extraction.
Jung et al. [29]	A randomized, controlled clinical trial to evaluate a new membrane for guided bone regeneration around dental implants	The aim of this study was to evaluate a synthetic resorbable hydrogel membrane based on polyethylene glycol (PEG) that could provide vertical bone fill comparable to that of a standard collagen membrane.	37	6 months	Vertical bone gain after 6 months was similar between PEG and collagen membranes (5.63 mm vs. 4.25 mm). PEG showed slightly more soft tissue issues but allowed for simplified application.	More soft tissue complications with PEG membrane (e.g., delayed/incomplete healing) but allowed for simplified application. All complications were resolved uneventfully.	PEG membrane achieved comparable regenerative outcomes to collagen with easier handling despite minor soft tissue complications.
Zwahlen et al. [4]	A comparison of two resorbable membrane systems in bone regeneration after the removal of wisdom teeth: a randomized controlled clinical pilot study	Randomized, prospective, and partially blinded pilot study, 15 patients received biodegradable membrane system Inion GTR™ on one side and the double-layer resorbable membrane Bio-Gide^®^ by Geistlich on the other.	15	3–6 months	Bone biopsies showed similar new bone formation between Inion (synthetic) and Bio-Gide (xenogenic) membranes. CT and histological analysis revealed no statistically significant differences in bone regeneration or membrane performance.	Mild adverse events (wound infection, hematoma, late swelling) in three patients; healing generally uneventful.	Both membranes performed similarly in terms of safety and regenerative capacity. The main difference lies in the origin: synthetic vs. animal.
De Angelis et al. [30]	3D-Printable Biopolymers for Socket Preservation Technique: Soft Tissues Response: A Pilot Randomised Clinical Study	Randomized study on the healing of post-extraction sites with 3D-printed biomaterials vs. control group.	39	6 weeks	Both 3D-printed biopolymers (PLA+HA and PCL+β-TCP) significantly outperformed the open healing control group in soft tissue closure for posterior sockets (*p* < 0.05). Anterior sockets healed fully in all groups by 4 weeks, with no significant differences.	No complications reported. Healing was uneventful in all 39 patients.	3D-printed biopolymer membranes effectively promote soft tissue closure after extraction, particularly in posterior sites.
Saini et al. [31]	Assessment of combination techniques in enhancing the regenerative potential of tricalcium phosphate graft in treatment of infrabony periodontal defects	The aim of the present study was to evaluate and compare the clinical outcomes after reconstructive surgery using tricalcium phosphate (TCP) alone; TCP and root conditioning with citric acid (CA); and TCP, CA, and an oxidized regenerated cellulose (ORC) membrane.	39	6 months	All groups (TCP alone, TCP + CA, TCP + CA + ORC) showed significant improvements in PPD reduction, CAL gain, and defect fill. No significant intergroup differences.	No significant complications reported.	TCP combination therapies are at least as effective as TCP alone for infrabony defect regeneration.
Pradeep et al. [32]	Clinical effectiveness of autologous platelet-rich plasma and Peptide-enhanced bone graft in the treatment of intrabony defects	This study was conducted to compare the effectiveness of two regenerative techniques (autologous PRP plus ABM/P-15 versus autologous PRP alone) in the treatment of intraosseous defects in humans, analyzing clinical and radiological parameters.	28	9 months	PRP + ABM/P-15 showed statistically significant improvement in clinical and radiologic outcomes versus PRP alone. Greater bone fill observed on CT in the test group.	No significant complications reported.	PRP + ABM/P-15 is more effective than PRP alone for treating intrabony defects. Larger studies needed.
Machado et al. [33]	Randomized clinical study of injectable dextrin-based hydrogel as a carrier of a synthetic bone substitute	The aim of this randomized controlled clinical trial was to compare alveolar ridge preservation outcomes using a synthetic bone substitute alone or combined with a dextrin-based injectable hydrogel.	12	6 months	DEXGEL Bone showed greater granule resorption, better handling, and improved implant primary stability. Similar bone volume and density compared to control.	No local or systemic complications observed.	DEXGEL Bone is a safe and effective injectable carrier that enhances bone substitute handling and implant stability.
Kusirisin et al. [34]	Polycaprolactone versus collagen membrane and 1-year clinical outcomes: a randomized controlled trial	The aim of this randomized controlled clinical trial was to evaluate and compare the outcomes of guided bone regeneration using a bilayered polycaprolactone membrane versus a collagen membrane over a 1-year period following implant placement.	24	1 year	PCL membrane showed similar buccal bone thickness (BBT) and soft tissue dimensional change (STC) outcomes compared to collagen membrane; no statistically significant differences at 1-year follow-up.	Four early membranes exposed were found in the test group and three in the control group at 2 weeks after surgery. No other biological complications were seen during the study periods.	PCL membrane provides comparable outcomes to collagen membrane for GBR with simultaneous implant placement; further studies with larger sample size are needed.
Jung et al. [35]	Comparison of a polyethylene glycol membrane and a collagen membrane for the treatment of bone dehiscence defects at bone level implants—a prospective, randomized, controlled, multicenter clinical trial	The aim of this randomized controlled multicenter clinical trial was to evaluate and compare the clinical outcomes of guided bone regeneration using a polyethylene glycol membrane versus a collagen membrane in the treatment of bony dehiscence defects at bone level implants.	117	18 months	PEG and collagen membranes both resulted in vertical bone fill (59.7% PEG vs. 64.4% BG). The non-inferiority of PEG could not be demonstrated; MBL slightly increased in both groups.	Soft tissue complications occurred in both PEG and collagen groups, without significant differences.	Both membranes supported bone regeneration, but PEG could not be shown to be non-inferior to collagen.
Santana et al. [36]	Synthetic polymeric barrier membrane associated with blood coagulum, human allograft, or bovine bone substitute for ridge preservation: a randomized, controlled, clinical and histological trial	The aim of this randomized controlled clinical and histological trial was to assess the extent of alveolar ridge preservation following socket grafting with blood coagulum, human allograft, or bovine bone substitute, each covered by a synthetic polyethylene glycol membrane.	32	6 months	Use of AL + PEG membrane preserved ridge width (1.5 mm) better than BB (2.5 mm) or BC (2.3 mm). New bone formation was highest in BC (47.8%), followed by AL (33.3%) and BB (28.2%).	Post- surgical complications were minimal for all treatment modalities tested.	Alveolar bone preservation was best achieved using AL with PEG barrier. Different graft materials yielded different bone formation percentages.
Arunjaroensuk et al. [37]	The Stability of Augmented Bone Between Two Different Membranes Used for Guided Bone Regeneration Simultaneous with Dental Implant Placement in the Esthetic Zone	The aim of this randomized controlled clinical trial was to compare the effectiveness of a synthetic polylactic acid membrane versus a collagen membrane in maintaining buccal bone thickness following simultaneous guided bone regeneration and implant placement.	48	6 months	Both PLA (synthetic) and collagen membranes showed comparable reductions in facial bone thickness at all levels (0–6 mm apical to implant shoulder). No statistically significant differences.	Minor complications of gingival inflammation and membrane exposure were observed in three cases in the test group and two cases in the control group, but all sites recovered uneventfully.	PLA membrane is as effective as collagen membrane in maintaining stable augmented bone in the esthetic zone.

## Data Availability

Data are available upon request to the corresponding author.

## Abbreviations

**Abbreviation**	**Definition**
GBR	Guided Bone Regeneration
GTR	Guided Tissue Regeneration
RCT	Randomized Controlled Trial
PLA	Polylactic Acid
PCL	Polycaprolactone
PEG	Polyethylene Glycol
PRGF	Platelet-Rich Growth Factors
PRF	Platelet-Rich Fibrin
PRP	Platelet-Rich Plasma
TCP	Tricalcium Phosphate
MMP	Matrix Metalloproteinases
ORC	Oxidized Regenerated Cellulose
PDLA	Poly-D-Lactic Acid
ABM/P-15	Anorganic Bone Matrix/Peptide-15.
PRISMA	Preferred Reporting Items for Systematic Reviews and Meta-Analyses
PDL	Periodontal Ligament
CA	Citric Acid
